# Application of Raman Spectroscopy to Dynamic Binding Capacity Analysis

**DOI:** 10.1177/00037028231210293

**Published:** 2023-11-01

**Authors:** James W. Beattie, Ruth C. Rowland-Jones, Monika Farys, Hamish Bettany, David Hilton, Sergei G. Kazarian, Bernadette Byrne

**Affiliations:** 1 Department of Life Sciences, 4615 Imperial College London, London, UK; 2 Department of Chemical Engineering, 4615 Imperial College London, London, UK; 3 Biopharm Process Research, Medicine Development and Supply, GSK R&D, Stevenage, Hertfordshire, UK

**Keywords:** Biotherapeutic, monoclonal antibody, protein A affinity chromatography, dynamic binding capacity, Raman spectroscopy, high-performance affinity chromatography, HPAC

## Abstract

Protein A affinity chromatography is a key step in isolation of biotherapeutics (BTs) containing fragment crystallizable regions, including monoclonal and bispecific antibodies. Dynamic binding capacity (DBC) analysis assesses how much BT will bind to a protein A column. DBC reduces with column usage, effectively reducing the amount of recovered product over time. Drug regulatory bodies mandate chromatography resin lifetime for BT isolation, through measurement of parameters including DBC, so this feature is carefully monitored in industrial purification pipelines. High-performance affinity chromatography (HPAC) is typically used to assess the concentration of BT, which when loaded to the column results in significant breakthrough of BT in the flowthrough. HPAC gives an accurate assessment of DBC and how this changes over time but only reports on protein concentration, requires calibration for each new BT analyzed, and can only be used offline. Here we utilized Raman spectroscopy and revealed that this approach is at least as effective as both HPAC and ultraviolet chromatogram methods at monitoring DBC of protein A resins. In addition to reporting on protein concentration, the chemical information in the Raman spectra provides information on aggregation status and protein structure, providing extra quality controls to industrial bioprocessing pipelines. In combination with partial least square (PLS) analysis, Raman spectroscopy can be used to determine the DBC of a BT without prior calibration. Here we performed Raman analysis offline in a 96-well plate format, however, it is feasible to perform this inline. This study demonstrates the power of Raman spectroscopy as a significantly improved approach to DBC monitoring in industrial pipelines.

## Graphical abstract

**Graphical abstract:**
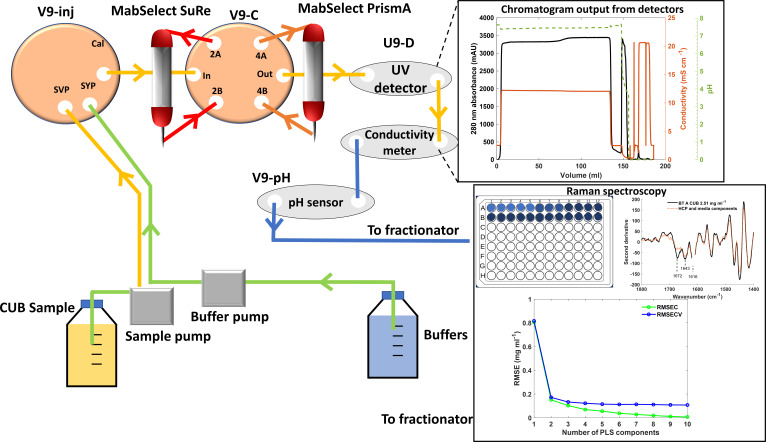
This is a visual representation of the abstract.

## Introduction

Biotherapeutics (BTs) are bioactive, recombinantly produced molecules that are increasingly widely used for the treatment of a range of chronic and acute conditions. They include a range of different proteins and although monoclonal antibodies (mAbs) are currently the most widely used, there are others including bispecific antibodies and growth factors that are also commonly prescribed.^1,2^ A total of 175 mAbs were approved for the market as of 2021,^1,3^ for the treatment of diseases including coronavirus infection,^3^ and chronic lymphoblastic leukemia.^4,5^ The best-selling therapeutics of 2019 were all mAbs, with the top nine having cumulative sales of US$75 billion.^6^ Other BTs, such as bispecific antibodies^1^ and fusion proteins,^2^ are also beginning to be more widely used, with four and twelve currently commercially available, respectively. As each BT and the cell line used to produce it are unique, the isolation protocol needs to be optimized in each case. In addition, in order to meet regulatory requirements, there is a need to remove a wide range of contaminants from the feed stream containing the expressed BTs.^7^ The development of bespoke isolation protocols and the requirement for expensive affinity resins (protein A resin costing up to $14 000 per liter) contributes substantially to the cost of BTs.^8^

The most widely used downstream processing step, protein A chromatography, removes the bulk of contaminants from the feed stream.^9^ Analysis of the ability of the protein A resin to bind antibody can be assessed in a number of ways including static binding capacity and attenuated total reflection Fourier transform infrared spectroscopy.^10^ In order to use the protein A resin as efficiently as possible and thus reduce operational costs, dynamic binding capacity (DBC) analysis is performed. DBC determines how much BT can bind to a column before a set percentage (e.g., 10%) of the product is lost as breakthrough.^11^ The main experimental parameter to be changed during these assays is the residence time of BT in the column, controlled by the flow rate. Due to the fact that BT binding is driven by mass transport phenomena,^11^ a longer contact time means a higher chance of the BT binding to the protein A ligand. There is however a tradeoff between unit operation time and overall BT binding to the column and as such, there will typically be a residence time beyond which only minimal gains in DBC will be achieved. The performance of columns after repeated use for each BT is mandated by the U.S. Food and Drug Administration.^7^ The DBC of protein A resin for a given BT is normally assessed in industry using HPAC due to its sensitivity to small amounts of analyte. In addition, real-time monitoring approaches, such as ultraviolet (UV) spectroscopy, have the advantage of allowing rapid adaptation of a step during continuous processing. An example is the utilization of ÄKTA purification systems, which come with a built-in UV flow cell for protein quantification.

However, both approaches report only the concentration of protein^11–14^ and the HPAC analysis can only be carried out offline. In order to make the process of monitoring DBC and other BT attributes as effective and efficient as possible, and reduce the cost of the BTs, it is important that alternative methods are explored. Raman spectroscopy in combination with multivariate methods is of particular interest as it reduces the need to carry out multiple orthogonal methods to obtain information on a range of protein parameters, e.g., protein concentration, aggregation status, and protein structure.

Raman spectroscopy is a powerful analytical tool enabling both qualitative and quantitative analysis of vibrational modes where a change in polarizability occurs across a molecular bond. The measurement of the energy of photons that are inelastically scattered by a sample is known as the Raman shift and this value corresponds to a frequency of vibrational modes of a molecule.^15^ The vibrational modes provide information on the chemical makeup of a sample. In addition, the secondary structure of any specific protein sample can be determined.^16,17^ Raman spectroscopy has already demonstrated the ability to determine and quantify multiple characteristics or product quality attributes of a given isolated BT including, protein stability in particular formulations (e.g., pH/additives), aggregation status of lyophilized BT and glycosylation status.^18–20^ Raman spectroscopy has also been successfully used in pilot scale bioreactors for monitoring both glycosylation of the expressed protein and glucose concentration of the media.^21,22^ Recently, Raman spectroscopy using a fiber optic ball probe was successfully used to quantify mAb titer during the loading of a protein A column.^23^ This inline Raman method did not perform as well as a UV-based approach and the authors recommended one way to optimize the approach would be to utilize simple regression techniques such as partial least square (PLS) rather than the convolutional neural networks they applied.^23^ Raman spectroscopy has also been utilized in other areas of downstream processing, including quantification of both monomer and aggregate during polishing steps such as cation exchange chromatography, however, when buffer salt concentration was beyond that of the training model prediction accuracy was significantly decreased.^24^

Previous research from our group utilized Raman spectroscopy to explore how column use affects mAb binding to protein A resin and provides insights into the causes of resin fouling.^25^ Here, we have assessed the ability of Raman spectroscopy to establish both BT titer and DBC of two different, widely used protein A affinity resins, i.e., MabSelect SuRe (MSS) and MabSelect PrismA (MSP), Cytiva Life Sciences. Raman spectroscopy achieved results comparable to industry standard methods such as UV monitoring during chromatography and HPAC with the added advantage of producing multivariate data which has the potential to be further developed to quantify multiple protein characteristics crucial for BT quality and homogeneity such as host cell protein (HCP) titer, glycosylation status, and protein stability simultaneously.

## Materials and Methods

### Biotherapeutic Preparation

Unprocessed bulk (UPB) solutions obtained from individual pilot scale bioreactor cultures of BTs A, B, and C were provided by GSK. For confidentiality reasons, we are not able to specify the precise nature of the BTs used in this study. BT A and BT B were used for all analyses. BT C was only used for purification cycles 12–21 and subsequent HPAC analysis (Table S1, Supplemental Material). Each BT is part of an active medicine development program at GSK and contains a fragment crystallizable region required for protein A affinity chromatography. Each UPB sample was defrosted at room temperature as required and then passed through a 0.2 µm bottle top filter (Merck) to remove particulate matter and produce clarified unprocessed bulk (CUB) solution. This clarification process was performed in a sterile laminar airflow hood using aseptic techniques to prevent microbial growth. CUB samples were aliquoted into a mix of 1 and 5 L containers. The 1 L CUB aliquots were stored at 4 °C for up to five days while 5 L CUB aliquots were frozen at –80 °C until further use.

### Generation of BT Breakthrough Curves

The HiScreen MSS and HiScreen MSP columns were attached to the valves and tubing of an ÄKTA Avant 25 (Cytiva) system as illustrated in (Figure S1, Supplemental Material), in order to ensure that the offset delay volume for DBC calculations remained the same through all experiments. Both columns were equilibrated with three column volumes (CVs) of equilibration buffer and then loaded with CUB to at least twice the column DBC stated by the manufacturer (35 mg/mL for MSS at a 2.4 min residence time^26^ and 65 mg/mL for MSP at a 4 min residence time^27^) so the column was oversaturated as calculated using Eq. 1:

(1) $$C_{S}=\frac{V_{I}\;\times C_{0}}{V_{c}}\$$$

where C_S_ is the oversaturation concentration, V_I_ is the injection volume, C_0_ is the BT titer, and V_c_ is the column bed volume.

During each protein loading step, 2 mL fractions were collected in a 96-deep well plate maintained at 4 °C. The flow rate was varied between runs, allowing for control of the residence time of BT during each run (flow rates of 0.47, 0.78, and 0.93 mL/min correspond to residence times of 2, 4, and 5 min, respectively). The columns were subsequently washed with three CVs of equilibration buffer to remove unbound contaminants and the bound BT was eluted with elution buffer (1.8 mM sodium acetate, 28.2 mM acetic acid, pH 3.6). The columns were washed with three CVs of MilliQ water followed by three CVs cleaning-in-place (CIP) buffer (0.1 M NaOH) with a 15 min CIP buffer hold on the column. Once runs were completed the columns were stored in 20% ethanol solution at 4 °C.

### Obtaining Breakthrough Titers Using HPAC

In order to calculate the titer of BT in the breakthrough, a 1260 infinity II (Agilent) high-performance liquid chromatography (HPLC) system fitted with a 20 µm POROS (Thermo Scientific) protein A column was used. A bind-and-elute mode was used. The flow rate was 2 mL/min, and the column/sample compartments were all maintained at 5 °C. A detection wavelength of 280 nm was used. The area under the elution peak for each isolated BT was measured and compared to a BT standard curve using Eqs. 2 and 3:

(2) $$C_{M}=\frac{S_{A}-b}{m}\;$$

where C_M_ is the mass on column, S_A_ is the peak area, m is the standard curve gradient, and b is the standard curve *y*-intercept, Then,

(3) $$C_{0}=\frac{C_{M}}{I_{V}}$$

where I_V_ is the injection volume and C_0_ is the BT titer in which C_M_ is the mass on column, S_A_ is the peak area, m is the standard curve gradient, and b is the standard curve *y*-intercept. The calculated C_M_ was then used in $C_{0}=C_{M}/I_{V}$ , where I_V_ was the injection volume to obtain C_0_, the BT titer*.*

Standard curves were generated by diluting BT solutions at concentrations of 1.00 mg/mL (±0.02) for BT A and 0.96 mg/mL (±0.01) for BT B. The standard solution concentration for each BT was verified by OD_280_ nm measurements on a Lunatic UV visible absorbance spectrometer (Unchained Labs) using ε values of 1.44 and 1.46 for BT A and B, respectively (*n* = 3). Each BT solution was placed into the multisampler of the 1260 infinity II (Agilent) HPLC system. The POROS column was equilibrated with 50 mM sodium phosphate and 0.5 M NaCl (pH 7.0), which was then loaded with a range of set volumes (5, 7, 10, 15, 20, 25, and 30 µL) of each BT solution. The bound protein was eluted with 50 mM sodium phosphate, 0.5 M NaCl, pH 2.0. Elution of BT was confirmed by the integrated UV detector. Upon determination of the BT titer by HPAC, Eq. 4 was utilized to calculate the DBC:

(4) $$\mathrm{DB}C_{10\text{\%}}=\frac{(V_{10\text{\%}}-V_{0})\;\times C_{0}}{V_{c}}$$

where DBC_10%_ is the DBC at 10% breakthrough, V_10%_ is the volume at 10% breakthrough, V_0_ is the offset volume, V_c_ is the column bed volume, and C_0_ is the BT titer.

### High-Throughput 96-Well Plate Raman Microscopic Analysis

The InVia confocal Raman system's (Renishaw) stage was optically focused using a pen mark on the bottom of the transparent quartz 96-well plate. Once the mark was in focus the stage was set 5000 µm from the objective in the *z*-direction. A quartz 96-well plate containing 200 µL aliquots of individual breakthrough samples was used for Raman microscopy. A plate mapping add-on was used to map the 96-well plate ensuring that all sample wells were analyzed. A 10× objective was used with the pinhole in the out position. The 785 nm laser set to 500 mW power, 20 accumulations, an exposure time of 24 s, and a 1200 grooves/mm grating was used in combination with the built-in cosmic ray removal setting. Samples were allowed to be in the well plate for a maximum of 4.5 h in order to minimize sample evaporation. Over this time scale, the sample showed no discernable reduction in quality as assessed by analysis of variance of the amide I peak percentage obtained from repeated measurements of BT B (Figure S2, Supplemental Material). The generated spectra were exported to Matlab 2019B with PLS toolbox 9 (Eigenvector). Raman spectra were subjected to standard normal variate (SNV) normalization before further preprocessing and analysis within Matlab 2019B. Once the titer was calculated, Eq. 4 was utilized to determine DBC.

## Results and Discussion

### High-Performance Affinity Chromatography (HPAC) Versus UV Chromatographic Analysis of DBCs

The DBC samples were generated using BT A and BT B, with different resins (MSS or MSP) or residence times. The samples were designated as “Train” or “Test” for subsequent PLS analysis (Table I). Train refers to samples used in the generation of PLS Raman models (see below) whereas test samples were used to test the model for predictive ability for the same BT at different residence times or for different BT. Training data samples were generated from BT A at residence times of 2 min and 5 min. Test data were generated for both BT A and BT B at a residence time of 4 min (Table I). This range of residence times is similar to that explored by previous DBC studies^11,13,28^ and manufacturer testing of MabSelect type resin.^27^

**Table I.** HPAC and UV chromatogram calculated DBC for each HiScreen column, BT, and residence time over purification cycles 26–29.

HiScreen resin type	Cycle	BT	Residence time (min)	HPAC DBC (mg/mL)	UV chromatogram (mg/mL)	Sample designation
MabSelect SuRe	26	A	5	38.94	37.69	BT A Train
MabSelect SuRe	27	A	2	27.64	29.49	BT A Train
MabSelect SuRe	28	A	4	36.97	39.23	BT A Test
MabSelect SuRe	29	B	5	37.45	36.54	BT B Test
MabSelect PrismA	26	A	5	61.27	63.91	BT A Train
MabSelect PrismA	27	A	2	41.37	44.26	BT A Train
MabSelect PrismA	28	A	4	58.63	61.98	BT A Test
MabSelect PrismA	29	B	5	59.21	56.62	BT B Test

Samples were divided into training and test samples for subsequent Raman analysis based on BT type.

**Figure 1.**
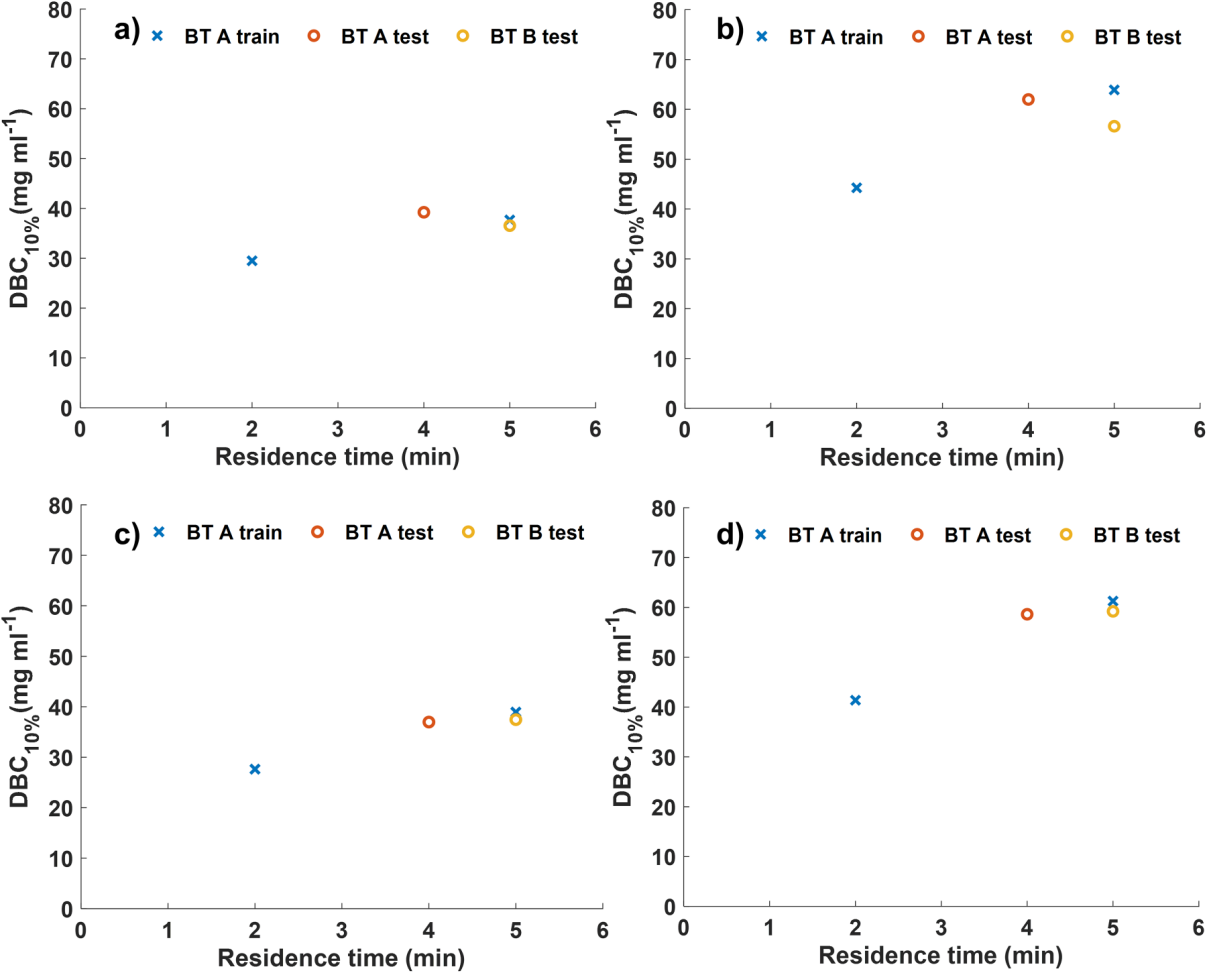
High-performance affinity chromatography (HPAC) and UV chromatogram calculated DBCs of HiScreen columns over cycles 26–29. DBC results for breakthrough samples used in subsequent Raman analysis. (a) UV calculated DBC results for MSS, (b) UV calculated DBC for MSP, (c) HPAC calculated DBC for MSS, and (d) HPAC calculated DBC for MSP. Blue crosses represent BT B at residence times of 2 min and 5 min (BT B train). Red circles represent BT B at a residence time of 4 min (BT B test). Yellow circles represent BT A at a residence time of 5 min. This figure shows only data used for training and testing the Raman PLS model, triplicate measurements of BT A at residence times of 2 min and 5 min are shown in Figure S3 (Supplemental Material).

The breakthrough curves for both MSS and MSP resins were generated over 30 cycles and then four were selected (cycles 26–29) to assess the DBCs at different residence times (Table I and Figure 1) using both HPAC and UV chromatography approaches. Very similar DBCs are obtained with HPAC (Figures 1a and 1b) and UV chromatography (Figures 1c and 1d) and in both cases, the DBC increases with increased residence time (Table I). Both approaches reveal that MSP has an increased DBC for BT A and BT B compared to MSS, as expected (i.e., Cytiva^27^ and Pabst et al.^28^). The DBCs obtained with MSP were higher by between ∼14 and 22 mg/mL depending on the residence time.

Overall column performance, assessed in terms of DBC, decreased as cycle number increased, likely due to irreversible binding of HCP contaminants.^25^ An example of this column degradation is seen for MSP with BT B between cycle 6 (Table S1, Supplemental Material) and cycle 29 (Table I) where a 4% and 5% decrease in DBC is observed for the UV chromatogram and HPAC methods, respectively.

### Raman Spectroscopy Analysis of Samples

**Figure 2.**
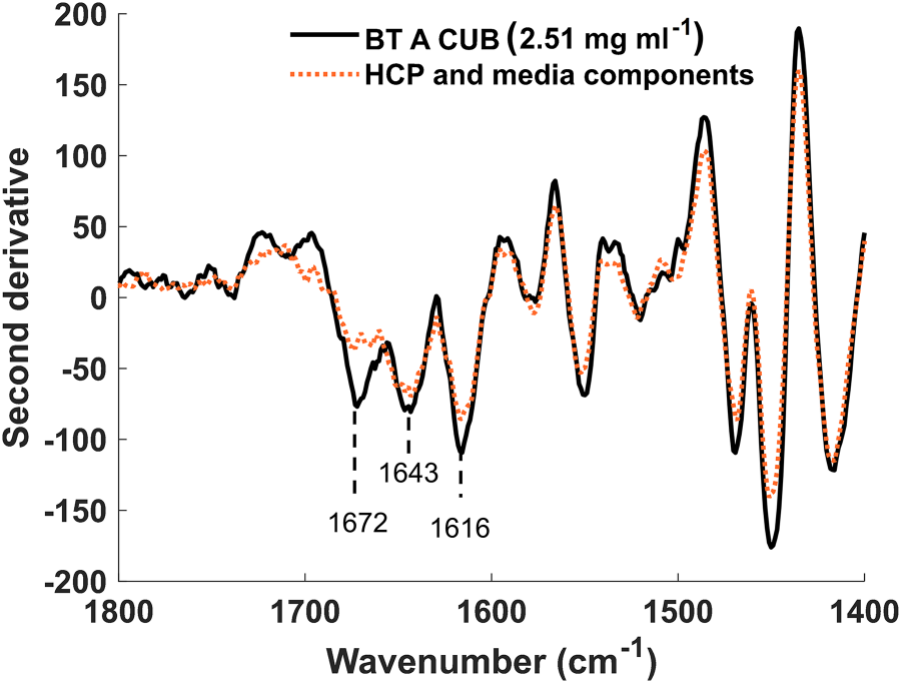
Second-derivative Raman spectrum showing the difference in the CUB spectrum when BT A is present. The spectrum was rubber band baseline corrected. SNV was normalized and Savitzky–Golay filtered (window = 27, derivative = 2, and polynomial = 2). The spectrum obtained for BT A CUB flowthrough from the MSS column, containing HCPs and media components is shown in the orange dotted line. The absence of the BT A from this sample was confirmed by HPLC. The spectrum obtained for CUB containing BT A at 2.51 mg/mL is shown in black. Amide I representative peaks are labeled, and the 1672 cm^–1^ represents the β-sheet structure present in BT A.

The samples analyzed by HPAC were also analyzed by Raman spectroscopy. By normalizing band intensities, a three-peak analysis of the amide I band can be used to monitor the protein structure; peak one (1657–1688 cm^–1^) representing β sheet structures, peak two (1630–1656 cm^–1^) representing α-helical structures and peak three (1598–1630 cm^–1^) representing phenylalanine and other aromatic residues in the protein tertiary structure (Figure 2).

There is a sharp increase in normalized intensity for the amide I band when BT concentration increases. The peak representing β sheet structure at 1672 cm^–1^ shows the largest difference with increasing BT concentration. This difference in normalized intensity for the amide I band shows that differences in concentration were observable and thus BT could be quantified by a combination of Raman and PLS.

The resultant spectra from BT A “Train” flowthrough samples were used to train the simple PLS algorithm. The ideal number of PLS components was chosen using root mean squared error (RMSE) values (Eq. 5) to ensure overfitting did not occur.

(5) $$\mathrm{RMSE}\;=\;\;\sqrt{\frac{\sum_{i\;=\;1}^{n}\;{\left(y_{i}-{\hat{y}}_{i}\right)}^{2}}{n}}$$

where RMSE is the root mean square error, *n* is the number of observations, $y_{i}$ is the *i*th iteration of actual observation, and ${\hat{y}}_{i}$ is the *i*th iteration of predicted observation.

The RMSE values for individual models were determined based on actual values obtained from the HPAC-derived titer measurements and predicted Raman PLS-derived BT titers. This was possible as both HPAC and Raman used the same 2 mL fractions obtained from the DBC experiments.

In order to identify the ideal number of PLS components required to prevent overfitting, a model was generated by selecting the number of PLS components where no further gain in RMSE of cross-validated data (RMSECV) is observed. When the increase in RMSECV becomes incremental, the Q^2^, known as the goodness of prediction, can be used where 1 = perfect prediction and 0 = no predictive ability. Q^2^ was derived from cross-validating the data using leave-one-out cross-validation (LOOCV) (Table S2, Supplemental Material).

**Figure 3.**
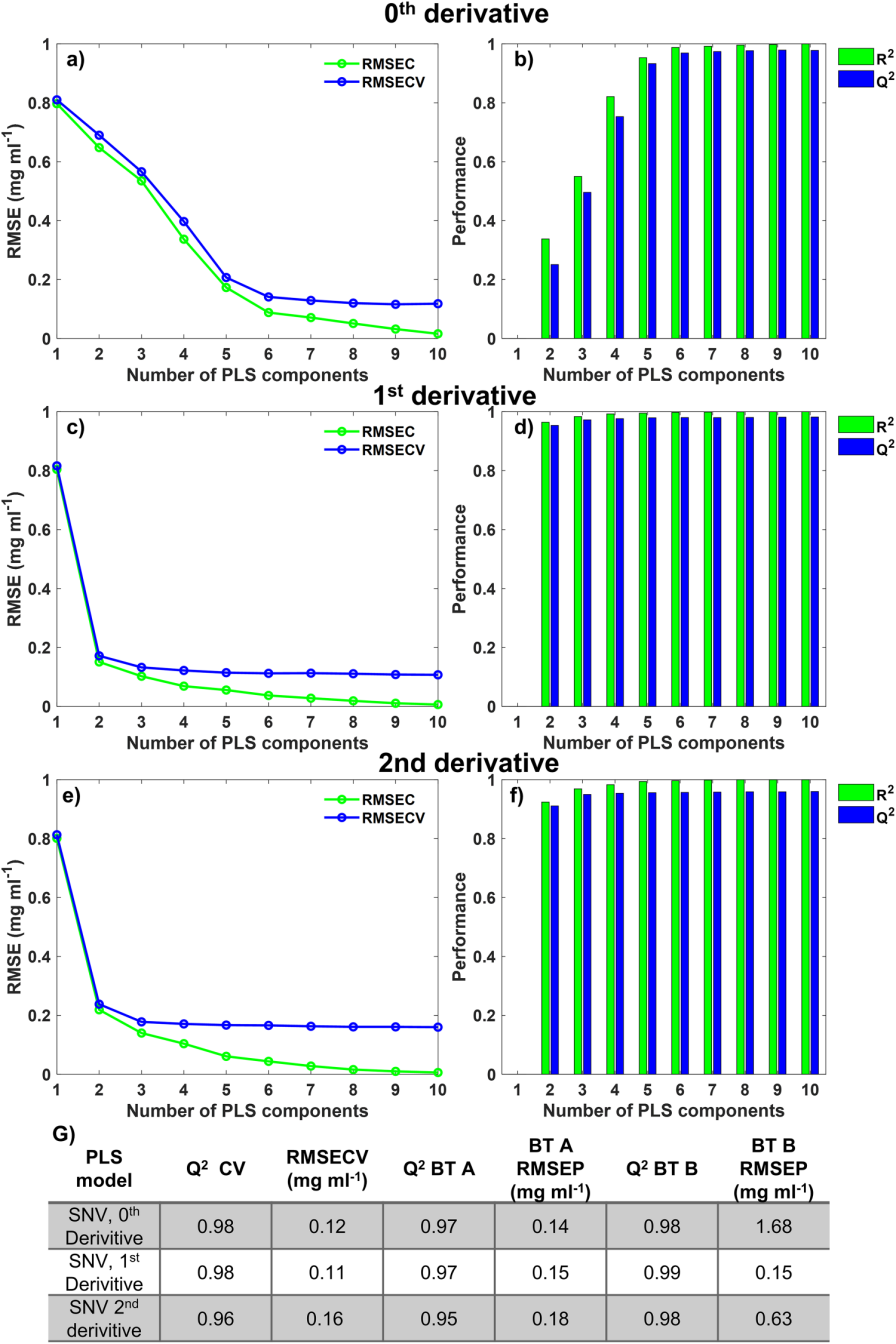
Statistical analysis of the Raman PLS model using spectral preprocessing for zeroth-, first- and second-derivative spectra. (a) RMSE plot for training data and LOOCV for SNV zeroth-derivative PLS model, (b) goodness of fit (R^2^) and goodness of prediction (Q^2^) plot for training data and LOOCV for the SNV zeroth-derivative PLS model, (c) RMSE plot on training data and LOOCV for SNV first-derivative PLS model, (d) R^2^ and Q^2^ plot for training data and LOOCV SNV first-derivative PLS model, (e) RMSE plot on Training data and LOOCV for SNV, second-derivative PLS model, and (f) R^2^ and Q^2^ plot for training data and LOOCV for SNV, second-derivative PLS model. Green = data obtained from applying the model to training data. Blue = data obtained from applying the model to LOOCV data. (g) RMSEP and Q^2^ for each Raman PLS model when applied to cross-validation, for both BT A and BT B.

The zeroth-order model with SNV normalization at eight components had an RMSE of 0.120 mg/mL and Q^2^ of 0.977 (Figures 3a and 3b), whereas the eight-component, second-derivative SNV normalized model had an RMSE of 0.161 mg/mL and a Q^2^ of 0.959. The SNV and Savitzky–Golay filter (window = 25, derivative = 2, and polynomial = 2) preprocessed model was chosen to be an eight-component model (Figures 3e and 3f). The first-order model with SNV and Savitzky–Golay filter (window = 15, derivative = 1, and polynomial = 1) performed best in cross-validation with an RMSE and Q^2^ of 0.112 mg/mL and 0.980, respectively, for a six-component system (Figures 3c and 3d). Of the three optimized Raman PLS models for the quantification of BT, the lower RMSE and higher Q^2^ showed the first-derivative model to be best for the quantification of BT in flowthrough samples. The predictive ability of the model was assessed by the RMSE of prediction (RMSEP). The RMSEP is generated when the RMSE for the model is applied to “Test” data sets, in this case, BT A and BT B test data as defined in Table I.

The SNV first-derivative preprocessing also yielded the second-lowest RMSEP for BT A-based models. However, it was the only model to achieve RMSEP below 0.2 mg/mL when applied to the different BT, BT B. This first-derivative PLS model is optimal for a platform approach, allowing analysis of new BTs without any associated BT calibration as required for HPAC analysis. The quantification of BT titer in individual BT flowthrough fractions and CUB by Raman spectroscopy meant the DBC for each test DBC experiment could be derived according to Eq. 4.

### Comparison of the Different Methods

The ability of the HPAC, UV chromatogram, and the Raman spectroscopic methods to predict DBC prediction results were compared in terms of the coefficient of variation for a 5 min residence time of BT B for both HiScreen column types. For MSS, DBC predictions from cycles 5, 8, and 29 were used. For MSP DBC predictions from cycles 6, 9, and 29 were used. The average percentage coefficient of variation (%CV) for both columns was calculated for both HPAC and UV chromatogram techniques using Eq. 6:

(6) $$\text{\%}\mathrm{CV}(\mathrm{SD})=\frac{\mathrm{SD}}{\bar{y}}\;\times 100$$

where $\bar{y}$ is the average actual measurement and SD is the standard deviation.

The UV chromatogram approach achieved a lower %CV of 2.7% compared to HPAC with a %CV of 7.1%, indicating that the UV chromatogram is a more precise approach for obtaining DBCs. As a result, the Raman DBC prediction method was compared to chromatogram-derived DBC values using Eq. 7:

(7) $$\text{\%}\mathrm{CV}(\mathrm{RMSE})=\frac{\mathrm{RMSE}}{\bar{y}}\;\times 100$$

where $\bar{y}$ is the average actual measurement.

The Raman-derived DBC had a %CV (RMSE) of 0.74%. When we performed the same comparison of the UV chromatogram obtained DBCs with those obtained by HPAC, this yielded a %CV (RMSE) of 1.25%. Thus, the Raman gave a slight reduction of 0.51% %CV (RMSE), indicating superior precision in assessing DBCs than the industry standard approach of HPAC. Importantly, for HPAC analysis, further processing of the sample is required in order to obtain the DBC. Here, we measured individual samples offline in a 96-well plate format, following fraction collection of the flowthrough, breakthrough, and eluate samples from the individual columns. However, it is certainly feasible to introduce an inline device containing a sapphire window, to allow Raman spectroscopic measurements of all fractions continuously in real-time with no sampling or further processing required. Sapphire is an ideal material as it only yields spectral peaks below 700 cm^–1^, and most BT peaks of interest are observed above 700 cm^–1^.

## Conclusion

As clearly demonstrated here, Raman spectroscopy is a viable alternative to the industry standard techniques, high-performance affinity chromatography (HPAC) and the UV chromatogram, for assessing the DBC of a protein A column for a given BT (Table II). Furthermore, in combination with PLS analysis, it can accurately predict the DBC for a new BT without the need for any BT-specific calibration, as shown by the DBC results obtained for BT B. The additional chemical information provided by Raman spectroscopy can provide extra insights into the structure and aggregation status of the protein as it elutes from the column. Clearly, in an industrial setting, these features are extremely useful as a means of ensuring batch-to-batch consistency of a given BT. Such information is not accessible through the current methods of HPAC and UV chromatogram methods of DBC determination. Utilizing Raman spectroscopy has the potential to provide better quality control of the sample and ultimately reduce the high cost of BTs to patients and health providers.

**Table II.** Summary of the DBCs obtained by the three different methods.

HiScreen resin type	Cycle	BT	RT^a^ (min)	HPAC DBC (mg/mL)	UV chromatogram DBC (mg/mL)	Raman DBC (mg/mL)
MabSelect SuRe	28	BT A test	4	36.97	39.23	38.48
MabSelect SuRe	29	BT B test	5	37.45	36.54	37.91
MabSelect PrismA	28	BT A test	4	58.63	61.99	60.42
MabSelect PrismA	29	BT B test	5	59.21	56.62	59.93

^a^ RT: residence time.

## Supplemental Material

sj-docx-1-asp-10.1177_00037028231210293 - Supplemental material for Application of Raman Spectroscopy to Dynamic Binding Capacity Analysis Supplemental material, sj-docx-1-asp-10.1177_00037028231210293 for Application of Raman Spectroscopy to Dynamic Binding Capacity Analysis by James W. Beattie, Ruth C. Rowland-Jones, Monika Farys, Hamish Bettany, David Hilton, Sergei G. Kazarian and Bernadette Byrne in Applied Spectroscopy
