# Supplementary material for: Application of Raman Spectroscopy to Dynamic Binding Capacity Analysis
Source: Appl Spectrosc. 2023 Nov 1;77(12):1393–400. doi: 10.1177/00037028231210293 (PMC10683347; doi:10.1177/00037028231210293)
Supplement: sj-docx-1-asp-10.1177_00037028231210293 - Supplemental material for Application of Raman Spectroscopy to Dynamic Binding Capacity Analysis [file sj-docx-1-asp-10.1177_00037028231210293.docx]

**Supplemental Material**

**Application of Raman Spectroscopy to Dynamic Binding Capacity Analysis**

James W. Beattie^1,2^, Ruth C. Rowland-Jones^3^†, Monika Farys^3^‡, Hamish Bettany^3^, David Hilton^3^, Sergei G. Kazarian^2^*, and Bernadette Byrne^1^*

^1^Department of Life Sciences, Imperial College London, SW7 2AZ, UK

^2^Department of Chemical Engineering, Imperial College London, SW7 2AZ, UK

^3^Biopharm Process Research, Medicine Development and Supply, GSK R&D, Gunnels Wood Road, Stevenage, Hertfordshire, SG1 2NY, UK

**Current addresses:**

†Lonza; 228 Bath Rd, Slough SL1 4DX

‡Polpharma Biologics S.A.; ul. Trzy Lipy 3, 80-172 Gdansk

*Corresponding author emails: s.kazarian@imperial.ac.uk; b.byrne@imperial.ac.uk


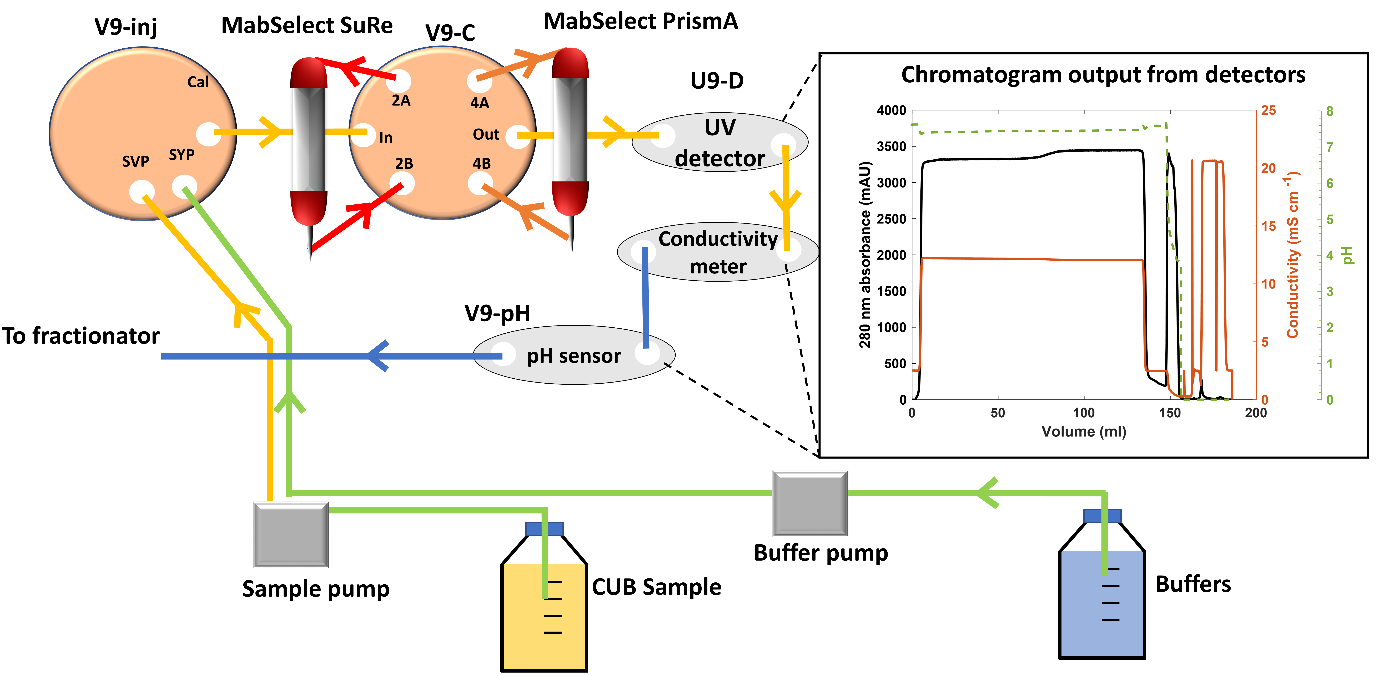


**Figure S1.** Schematic for the ÄKTA AVANT 25 purification system incorporating a HiScreen MSS (MAbSelect PrismA) column connected via ports 2A and 2B and HiScreen MSP (MabSelect SuRe) column connected via ports 4A and 4B were used to generate BT breakthrough curves. While the set up used is as presented here, only one of the columns was on-line during an individual experiment. From the V9 injection port the sample was diverted to the relevant column. Flowthrough, breakthrough, and eluate was pumped through the UV detector, yielding the chromatogram as shown, and then through the conductivity meter and pH sensor prior to collection in individual aliquots in the fractionator. The yellow pathway indicates the volume between the sample pump and conductivity meter (= system hold up volume). The red and dark orange pathways indicate the tubing and void volume of the HiScreen MSS and HiScreen MSP columns respectively. The void volume is the total volume in the column, i.e., between resin beads and within the pores of the resin beads. The offset volume is the yellow line plus the red/dark orange lines of either column.


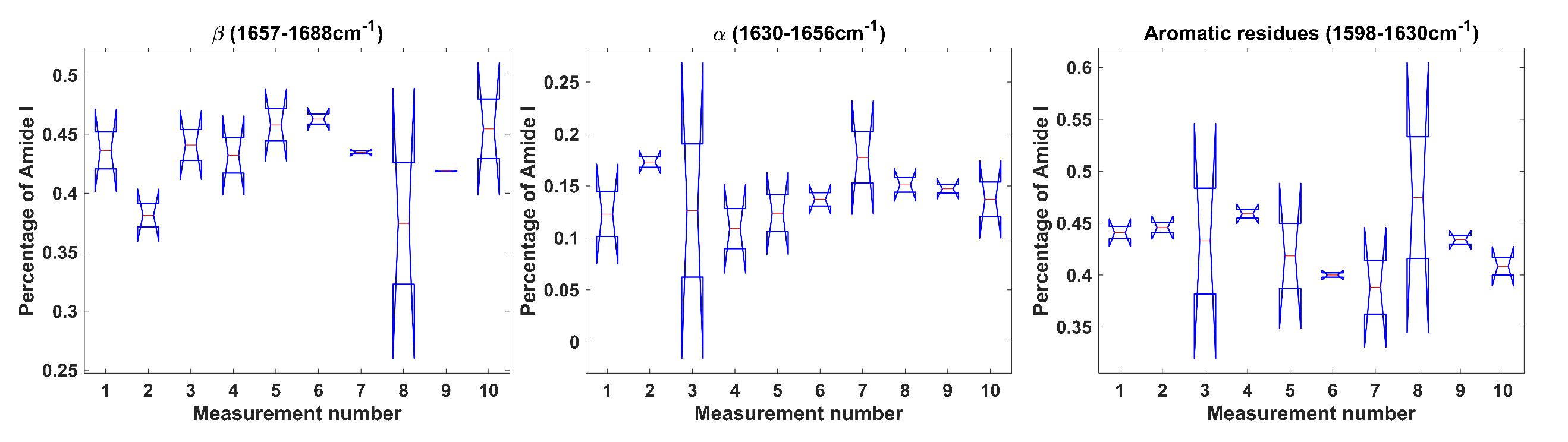
 **Figure S2****.** ANOVA box plots of the amide I peak percentage from Raman stability measurements of BT B CUB. Three amide I peaks representing A) β sheet structures (1667–1668 cm^–1^), B) α helical structures (1630–1656 cm^–1^) and C) aromatic residues (1598–1630 cm^–1^) were analysed (*n* = 2). Samples were measured 10 times with each measurement taking 19 min to acquire with no time interval between each measurement (total continuous laser exposure time of 190 min). The instrumental parameters of 20 accumulations, 24 s exposure, and 500 mW 785 nm laser power were used. The spectra for each of these amide I peaks (β, α, and aromatic residues) did not significantly change (*p* > 0.05) over the 10 repeated measurements with *p* values of 0.12, 0.64, and 0.55, respectively. There was no loss in specific amide I intensity measured indicating the sample was stable under these conditions and thus a 500 mW (100%) laser power was used for well plate measurements of samples.

**Table S1.** HPAC and ÄKTA UV Chromatogram calculated DBC for each HI screen column, BT, and residence time over purification cycles 1–25.

| HiScreen resin type | Cycle | BT | Residence time (min) | HPAC DBC  (mg ml^–1^) | UV Chromatogram  (mg ml^–1^) |
| --- | --- | --- | --- | --- | --- |
| MabSelect SuRe | 1 | B | 10 | 46.70 | 46.32 |
| MabSelect SuRe | 2 | B | 10 | 43.42 | 43.64 |
| MabSelect SuRe | 3 | B | 10 | 44.84 | 45.67 |
| MabSelect SuRe | 4 | B | 6 | 33.90 | 37.88 |
| MabSelect SuRe | 5 | B | 5 | 33.87 | 37.54 |
| MabSelect SuRe | 6 | B | 4 | 43.25 | 39.23 |
| MabSelect SuRe | 7 | B | 6 | 41.74 | 40.63 |
| MabSelect SuRe | 8 | B | 5 | 43.25 | 39.86 |
| MabSelect SuRe | 9 | B | 4 | 40.11 | 39.23 |
| MabSelect SuRe | 10 | B | 2 | 27.49 | 30.08 |
| MabSelect SuRe | 11 | B | 2 | 33.76 | 27.98 |
| MabSelect SuRe | 12 | C | 2 | 26.68 | 26.26 |
| MabSelect SuRe | 13 | C | 4 | 30.86 | 28.13 |
| MabSelect SuRe | 14 | C | 2 | 26.68 | 24.85 |
| MabSelect SuRe | 15 | C | 4 | 32.98 | 29.87 |
| MabSelect SuRe | 16 | C | 5 | 32.97 | 30.45 |
| MabSelect SuRe | 17 | C | 6 | 35.07 | 30.92 |
| MabSelect SuRe | 18 | C | 10 | 35.07 | 31.67 |
| MabSelect SuRe | 19 | C | 5 | 32.97 | 29.95 |
| MabSelect SuRe | 20 | C | 6 | 32.97 | 30.56 |
| MabSelect SuRe | 21 | C | 10 | 35.07 | 32.81 |
| MabSelect SuRe | 22 | A | 5 | 38.94 | 37.13 |
| MabSelect SuRe | 23 | A | 2 | 27.91 | 29.89 |
| MabSelect SuRe | 24 | A | 5 | 38.93 | 37.06 |
| MabSelect SuRe | 25 | A | 2 | 27.97 | 26.51 |
| MabSelect PrismA | 1 | B | 10 | 79.76 | 71.70 |
| MabSelect PrismA | 2 | B | 6 | 51.33 | 62.79 |
| MabSelect PrismA | 3* | B | 5 | N/A | N/A |
| MabSelect PrismA | 4 | B | 4 | 52.73 | 54.67 |
| MabSelect PrismA | 5 | B | 2 | 36.92 | 34.37 |
| MabSelect PrismA | 6 | B | 5 | 62.18 | 58.98 |
| MabSelect PrismA | 7 | B | 2 | 33.79 | 36.78 |
| MabSelect PrismA | 8 | B | 4 | 59.03 | 58.26 |
| MabSelect PrismA | 9 | B | 5 | 65.34 | 57.20 |
| MabSelect PrismA | 10 | B | 6 | 55.95 | 63.52 |
| MabSelect PrismA | 11 | B | 10 | 74.86 | 70.40 |
| MabSelect PrismA | 12 | C | 2 | 39.38 | 38.08 |
| MabSelect PrismA | 13 | C | 4 | 52.04 | 50.95 |
| MabSelect PrismA | 14 | C | 2 | 39.40 | 37.47 |
| MabSelect PrismA | 15 | C | 4 | 52.02 | 51.17 |
| MabSelect PrismA | 16 | C | 5 | 54.12 | 51.22 |
| MabSelect PrismA | 17 | C | 6 | 54.12 | 54.19 |
| MabSelect PrismA | 18 | C | 10 | 60.44 | 57.36 |
| MabSelect PrismA | 19 | C | 5 | 54.13 | 52.02 |
| MabSelect PrismA | 20 | C | 6 | 56.23 | 53.71 |
| MabSelect PrismA | 21 | C | 10 | 58.33 | 57.46 |
| MabSelect PrismA | 22 | A | 2 | 45.35 | 46.58 |
| MabSelect PrismA | 23 | A | 5 | 61.62 | 62.36 |
| MabSelect PrismA | 24 | A | 2 | 40.98 | 42.67 |
| MabSelect PrismA | 25 | A | 5 | 61.81 | 61.62 |

*Cycle 3 of Prism A exhibited a leak in the tubing to the column and thus the amount of mAb loaded was not accurate; The DBC for cycle 3 was not calculated by with Chromatogram or HPAC.

**Table S2.** statistical analysis of each PLS model between one and 10 components. Green highlighting denotes the chosen component number for prediction. SNV 0 derivative.

| No. Components | 1 | 2 | 3 | 4 | 5 | 6 | 7 | 8 | 9 | 10 |
| --- | --- | --- | --- | --- | --- | --- | --- | --- | --- | --- |
| RMSEC | 0.797 | 0.648 | 0.535 | 0.337 | 0.173 | 0.088 | 0.071 | 0.051 | 0.032 | 0.016 |
| R2 | 0.000 | 0.338 | 0.550 | 0.821 | 0.953 | 0.988 | 0.992 | 0.996 | 0.998 | 1.000 |
| RMSECV | 0.810 | 0.690 | 0.566 | 0.397 | 0.207 | 0.141 | 0.129 | 0.120 | 0.116 | 0.118 |
| Q2 | –0.031 | 0.251 | 0.496 | 0.753 | 0.933 | 0.969 | 0.974 | 0.977 | 0.979 | 0.978 |

**First-Derivative**

| No. Components | 1 | 2 | 3 | 4 | 5 | 6 | 7 | 8 | 9 | 10 |
| --- | --- | --- | --- | --- | --- | --- | --- | --- | --- | --- |
| RMSEC | 0.804 | 0.159 | 0.104 | 0.070 | 0.055 | 0.040 | 0.029 | 0.021 | 0.015 | 0.009 |
| R2 | –0.018 | 0.960 | 0.983 | 0.992 | 0.995 | 0.997 | 0.999 | 0.999 | 1.000 | 1.000 |
| RMSECV | 0.816 | 0.186 | 0.138 | 0.124 | 0.116 | 0.113 | 0.114 | 0.111 | 0.109 | 0.107 |
| Q2 | –0.048 | 0.946 | 0.970 | 0.976 | 0.979 | 0.980 | 0.980 | 0.981 | 0.981 | 0.982 |

**Second-Derivative**

| No. Components | 1 | 2 | 3 | 4 | 5 | 6 | 7 | 8 | 9 | 10 |
| --- | --- | --- | --- | --- | --- | --- | --- | --- | --- | --- |
| RMSEC | 0.801 | 0.219 | 0.140 | 0.104 | 0.061 | 0.044 | 0.028 | 0.016 | 0.010 | 0.006 |
| R2 | –0.009 | 0.924 | 0.969 | 0.983 | 0.994 | 0.997 | 0.999 | 1.000 | 1.000 | 1.000 |
| RMSECV | 0.813 | 0.238 | 0.178 | 0.171 | 0.167 | 0.166 | 0.163 | 0.161 | 0.161 | 0.160 |
| Q2 | –0.039 | 0.911 | 0.950 | 0.954 | 0.956 | 0.957 | 0.958 | 0.959 | 0.959 | 0.960 |


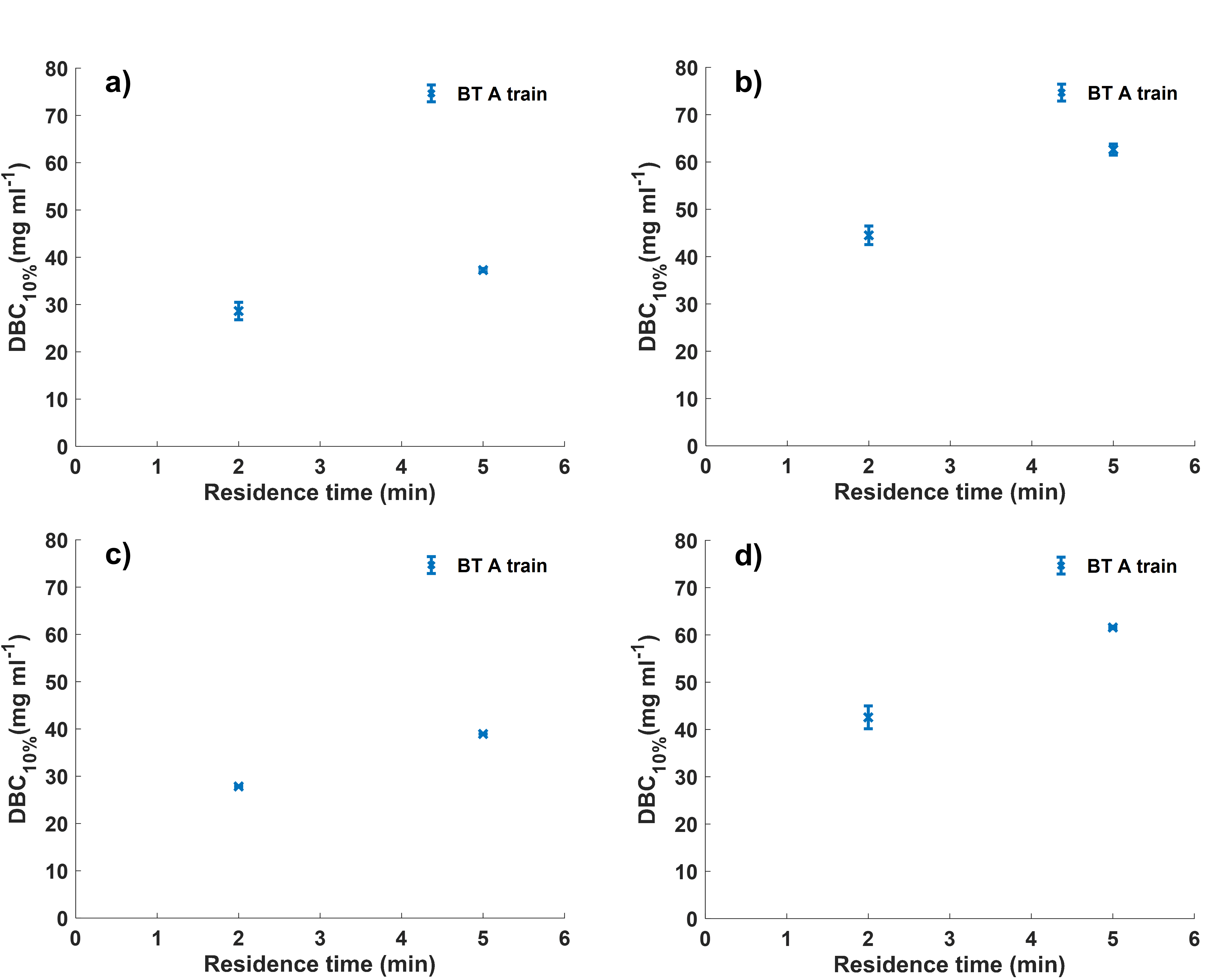

**Figure S3.** DBCs calculated for BT A at residence times of 2 min and 5 min. (a) UV calculated DBC results for MabSelect SuRe, (b) UV calculated DBC for MabSelect PrismA, (c) HPAC calculated DBC for MabSelect SuRe, and (d) HPAC calculated DBC for MabSelect PrismA; *n* = 3 with error bars representing the standard deviation.
